# 6β-Acetoxysandaracopimaradien-1α,9α-diol Attenuates LPS-Induced Acute Lung Injury: Association with Alterations in Src, MAPK, and Akt/GSK-3β Signalling

**DOI:** 10.3390/ijms27135969

**Published:** 2026-07-03

**Authors:** Nassareen Supaweera, Wanatsanan Chulrik, Chutima Jansakun, Aman Tedasen, Chuchard Punsawad, Porawan Pratumwan, Rungruedee Kimseng, Ratchanaporn Chokchaisiri, Apichart Suksamrarn, Warangkana Chunglok

**Affiliations:** 1 Health Sciences (International Program), College of Graduate Studies, Walailak University, Nakhon Si Thammarat 80160, Thailand; nassareen.sp@mail.wu.ac.th (N.S.); porawan.pr@mail.wu.ac.th (P.P.); 2 School of Allied Health Sciences, Walailak University, Nakhon Si Thammarat 80160, Thailand; wanatsanan.chu@wu.ac.th (W.C.); chutima.js@wu.ac.th (C.J.); aman.te@wu.ac.th (A.T.); 3 Food Technology and Innovation Research Center of Excellence, Walailak University, Nakhon Si Thammarat 80160, Thailand; 4 School of Medicine, Walailak University, Nakhon Si Thammarat 80160, Thailand; chuchard.pu@wu.ac.th; 5 Sanders Brown Center on Aging, University of Kentucky College of Medicine, Lexington, KY 40536, USA; kim.rrd@uky.edu; 6 Department of Chemistry, School of Sciences, University of Phayao, Phayao 56000, Thailand; ratchanaporn.ch@up.ac.th; 7 Department of Chemistry and Center of Excellence for Innovation in Chemistry, Faculty of Science, Ramkhamhaeng University, Bangkok 10240, Thailand; asuksamrarn@yahoo.com

**Keywords:** acute lung injury, isopimarane-type diterpenoid, network pharmacology, alveolar epithelial cells, inflammatory cytokines, pulmonary oedema

## Abstract

Experimental acute lung injury (ALI) models are widely used to investigate pulmonary inflammation and evaluate therapeutic strategies for acute respiratory distress syndrome (ARDS). *Kaempferia marginata* is a traditional medicinal plant used to treat fever and has been reported to possess anti-inflammatory properties in lipopolysaccharide (LPS)-activated macrophages. 6β-Acetoxysandaracopimaradien-1α,9α-diol (ASPD), a major isopimarane-type diterpenoid isolated from this plant, has not previously been investigated for its effects on ALI. This study employed an integrated network pharmacology, molecular docking, and experimental validation strategy to investigate the protective effects and potential mechanisms of ASPD against LPS-induced ALI. Network pharmacology analysis identified several inflammation-related hub targets associated with Src, MAPK, and PI3K/Akt signalling. In LPS-stimulated MLE-12 cells, ASPD reduced inflammatory cytokine production and inhibited the phosphorylation of JNK1/2, ERK1/2, p38 MAPK, Akt, and GSK-3β. In mice with LPS-induced ALI, ASPD alleviated histopathological lung injury, pulmonary oedema, and inflammatory cell infiltration while reducing IL-6, TNF-α, and myeloperoxidase activity without apparent toxicity. Immunohistochemical analysis demonstrated reduced Src and ERK1/2 expression in lung tissue. Molecular docking analysis predicted favourable binding affinities between ASPD and selected Src- and MAPK-related signalling proteins. These findings suggest that ASPD attenuates LPS-induced ALI and is associated with alterations in Src-, MAPK-, and Akt/GSK-3β-related signalling.

## 1. Introduction

Acute respiratory distress syndrome (ARDS) is a severe inflammatory lung disease associated with substantial morbidity and mortality worldwide [1]. Lower respiratory infections, particularly pneumonia, remain the leading infectious cause of death globally [2], and pneumonia is a major precipitating factor for ARDS in critically ill patients [3,4]. ARDS is characterised by diffuse alveolar damage, increased alveolar–capillary permeability, and severe impairment of gas exchange [4]. Despite advances in supportive care, effective pharmacological therapies for ARDS remain limited, and current treatments, including corticosteroids, demonstrate variable clinical efficacy [5], highlighting the need for novel therapeutic strategies targeting key inflammatory pathways.

Experimental acute lung injury (ALI) models, particularly lipopolysaccharide (LPS)-induced ALI, are widely used to investigate the pathophysiology of ARDS and evaluate potential therapeutic interventions because they reproduce several key inflammatory features of human ARDS, including neutrophilic infiltration, cytokine release, pulmonary oedema, and alveolar–capillary barrier dysfunction [6]. Since the alveolar epithelium serves as a key structural component of the alveolar–capillary barrier, preservation of epithelial integrity is crucial during lung injury and the progression of ARDS [7,8]. Alveolar epithelial type II (AT2) cells play an important role in ALI pathogenesis by amplifying inflammatory responses through interactions with immune cells, particularly macrophages and by producing cytokines [9,10]. In addition, AT2 cells contribute to alveolar repair and epithelial regeneration by serving as progenitor cells that proliferate and differentiate into alveolar epithelial type I cells following lung injury [11].

LPS stimulation activates Toll-like receptor 4 (TLR4)-associated signalling pathways, leading to the activation of multiple downstream inflammatory cascades [12]. Proto-oncogene tyrosine-protein kinase Src (Src) family kinases have been implicated in the regulation of inflammatory signalling during acute lung injury, including pathways associated with nuclear factor-kappa B (NF-κB) and mitogen-activated protein kinase (MAPK) signalling [13]. Activation of NF-κB promotes the transcription of pro-inflammatory cytokines, as demonstrated in LPS-stimulated alveolar epithelial cells [14], while MAPK signalling, particularly extracellular signal-regulated kinase (ERK), contributes to LPS-induced acute lung inflammation [15]. In addition, phosphoinositide 3-kinase (PI3K)/protein kinase B (Akt) signalling plays a key role in regulating inflammatory responses and lung epithelial injury [16]. Glycogen synthase kinase 3β (GSK-3β), a downstream target of Akt, has been identified as a pro-inflammatory mediator implicated in acute lung injury [17]. Furthermore, crosstalk between alveolar epithelial cells and alveolar macrophages modulates and enhances LPS-induced inflammatory responses in the lung [18,19]. These interconnected signalling pathways are implicated as potential molecular targets for therapeutic modulation in inflammatory responses associated with ALI [20].

Plants of the genus *Kaempferia* have long been used in traditional medicine for the treatment of respiratory disorders, including cough and asthma, with several species reported to exhibit expectorant, antitussive, and anti-inflammatory activities [21,22]. Among these, *Kaempferia marginata* is traditionally used as a fresh herbal ingredient and has been employed in Thai folk medicine for the treatment of fever [23,24]. Phytochemical studies have identified isopimarane-type diterpenoids as important secondary metabolites in several *Kaempferia* species [22]. 6β-Acetoxysandaracopimaradien-1α,9α-diol (ASPD), a major isopimarane-type diterpenoid isolated from *K. marginata*, has demonstrated anti-inflammatory activity by inhibiting nitric oxide production in LPS-stimulated macrophages [25] and suppressing key inflammatory mediators, including inducible nitric oxide synthase (iNOS) and cyclooxygenase-2 (COX-2), at the mRNA level [26]. These findings suggest that ASPD may modulate inflammatory signalling processes relevant to ALI; however, its role and underlying mechanisms in both in vitro and in vivo models of acute lung injury have yet to be elucidated.

Accordingly, the role of ASPD in inflammatory lung diseases, particularly ALI, remains poorly understood, especially with respect to its effects on key inflammatory mediators and signalling components. Therefore, this study aimed to investigate the protective effects of ASPD in LPS-induced ALI using complementary experimental approaches, including LPS-stimulated MLE-12 alveolar epithelial cells and a mouse model. Network pharmacology and molecular docking analyses were employed to identify potential molecular targets and signalling pathways associated with the effects of ASPD and to evaluate its potential interactions with selected target proteins.

## 2. Results

### 2.1. Network Pharmacology Analysis Predicts ALI-Related Targets and Pathways of ASPD

**Figure 1:**
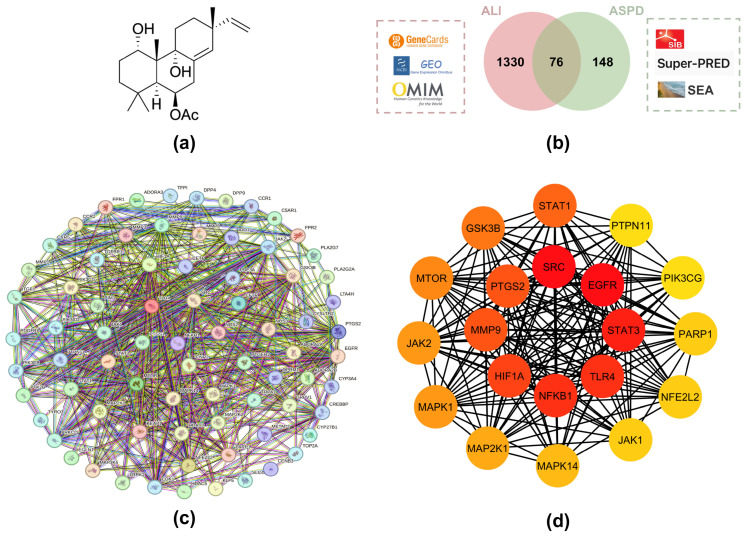
Network pharmacology analysis of ASPD in relation to acute lung injury (ALI). (**a**) The 2D chemical structure of ASPD; (**b**) Venn diagram showing the overlapping targets between ASPD and ALI (76 shared targets); (**c**) protein–protein interaction (PPI) network of the shared targets; (**d**) top 20 hub genes identified based on degree centrality. Node colours indicate degree centrality, ranging from red (highest) to yellow (lowest).

Network pharmacology analysis was performed to explore the potential molecular targets and pathways underlying the anti-ALI activity of ASPD (Figure 1a). SwissTargetPrediction, SuperPred, and the Similarity Ensemble Approach (SEA) collectively predicted 224 targets for ASPD. From the GeneCards, Gene Expression Omnibus (GEO), and Online Mendelian Inheritance in Man (OMIM) databases, 1406 ALI-related genes were retrieved, of which 76 overlapped with the predicted targets of ASPD (Figure 1b). These overlapping targets were subsequently used to construct a protein–protein interaction (PPI) network using the STRING database, resulting in a network comprising 76 nodes and 609 edges (Figure 1c). Topological analysis of the PPI network using Cytoscape identified 20 hub targets with the highest degree values, including SRC, EGFR, STAT3, TLR4, NFKB1, HIF1A, MMP9, PTGS2, STAT1, GSK3B, MTOR, JAK2, MAPK1, MAP2K1, MAPK14, NFE2L2, PARP1, JAK1, PIK3CG, and PTPN11 (Figure 1d and Table S1).

**Figure 2:**
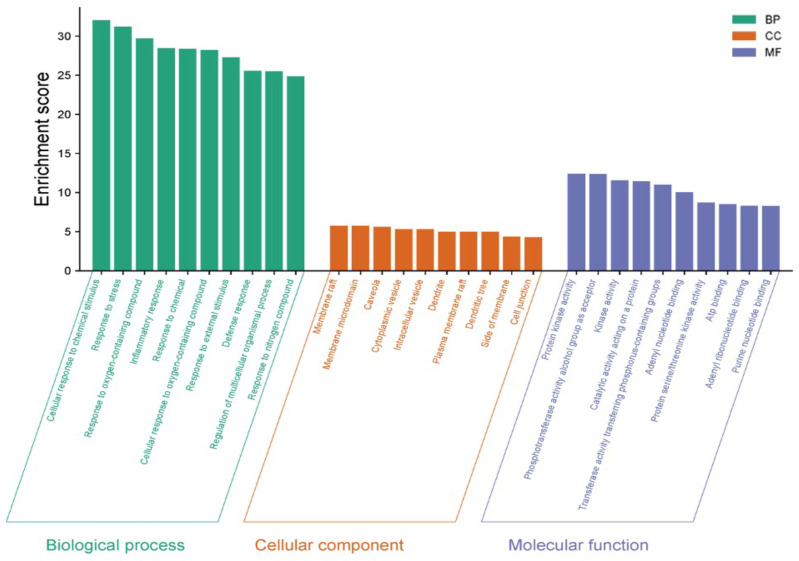
Gene Ontology (GO) enrichment analysis of ASPD-related targets. The diagram displays the enriched terms across biological processes, cellular components, and molecular functions.

**Figure 3:**
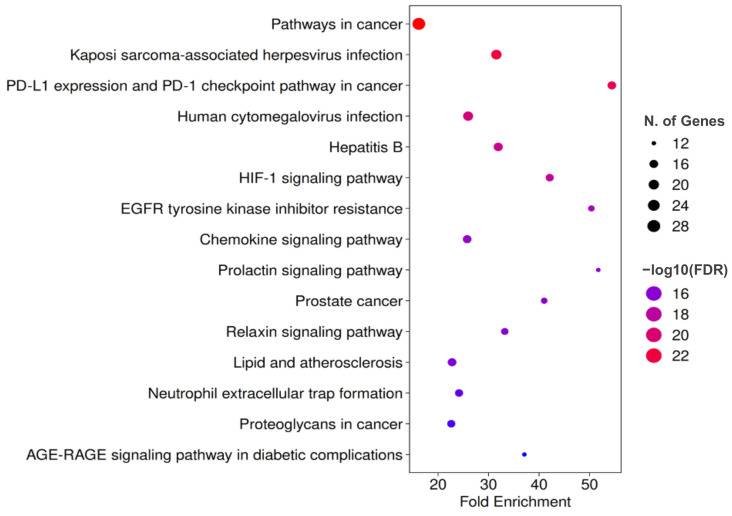
Kyoto Encyclopedia of Genes and Genomes (KEGG) pathway enrichment analysis. Fold enrichment and −log10 false discovery rate (FDR) values indicate the degree of enrichment and statistical significance, respectively. Dot size represents the number of genes involved in each pathway, while the colour gradient from blue to red represents increasing −log10(FDR) values, with red indicating higher statistical significance.

Gene Ontology (GO) enrichment analysis identified 1644 enriched terms, comprising 1000 biological process terms, 207 cellular component terms, and 437 molecular function terms. Among biological processes, the most enriched terms were primarily related to inflammatory/defence responses, stress responses, and cell migration (Figure 2 and Table S2). Enriched cellular components were primarily associated with membrane microdomains, including membrane rafts, plasma membrane rafts, and caveolae (Figure 2 and Table S3). Molecular function enrichment highlighted protein kinase activity, including protein serine/threonine kinase activity, protein tyrosine kinase activity, phosphotransferase activity, and transcription factor binding (Figure 2 and Table S4). Kyoto Encyclopedia of Genes and Genomes (KEGG) pathway enrichment analysis revealed 200 significantly enriched pathways, including the HIF-1 signalling pathway, chemokine signalling pathway, and neutrophil extracellular trap formation (Figure 3 and Table S5). These findings suggest that ASPD may interact with key protein targets associated with signalling pathways involved in ALI, supporting its potential role in modulating inflammatory responses. These predictions were subsequently evaluated through in vitro and in vivo experiments.

### 2.2. ASPD Reduces Inflammatory Cytokine Production in LPS-Stimulated MLE-12 Alveolar Epithelial Cells

**Figure 4:**
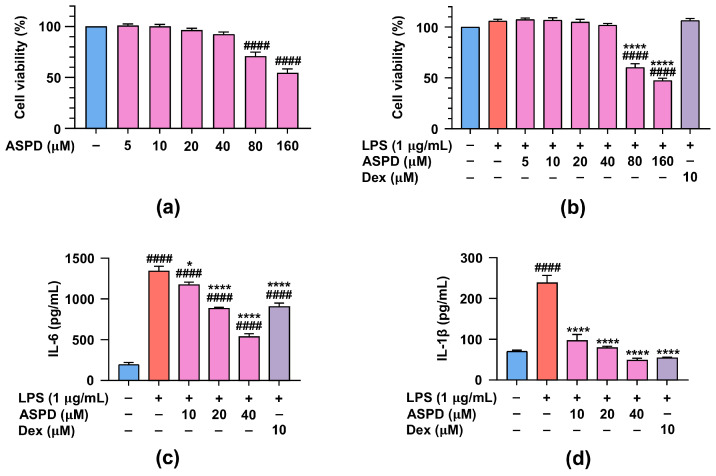
Effects of ASPD on cell viability and pro-inflammatory cytokine production in LPS-stimulated MLE-12 cells. (**a**) Cell viability following treatment with various concentrations of ASPD alone; (**b**) Cell viability of MLE-12 cells pretreated with ASPD in the presence of LPS (1 µg/mL), assessed by MTT assay; (**c**) IL-6 and (**d**) IL-1β production in LPS-stimulated MLE-12 cells treated with non-cytotoxic concentrations of ASPD. Dexamethasone (Dex, 10 µM) was used as a positive control. Data are presented as mean ± standard error of the mean (SEM) of three independent experiments. Statistical significance is indicated as follows: #### *p* < 0.0001 vs. the untreated control; * *p* < 0.05, **** *p* < 0.0001 vs. the LPS-treated group.

To investigate the cytotoxicity and anti-inflammatory effects of ASPD in vitro, cell viability was first evaluated using a 3-(4,5-dimethylthiazol-2-yl)-2,5-diphenyltetrazolium bromide (MTT) assay in MLE-12 alveolar epithelial cells. ASPD at concentrations of 5–40 μM did not exhibit cytotoxicity, maintaining cell viability above 80% under both basal conditions (Figure 4a) and LPS-stimulated conditions (Figure 4b).

Based on these results, ASPD concentrations of 10–40 μM were selected for subsequent experiments. LPS stimulation significantly increased the production of pro-inflammatory cytokines, including interleukin-6 (IL-6) and interleukin-1β (IL-1β), in MLE-12 cells (Figure 4c,d). Treatment with ASPD significantly reduced the levels of both cytokines in a concentration-dependent manner. Similarly, dexamethasone (Dex), used as a positive control, decreased cytokine production. These findings indicate that ASPD may exert anti-inflammatory effects in LPS-stimulated MLE-12 cells by reducing pro-inflammatory cytokine production.

### 2.3. ASPD Reduces MAPK and Akt/GSK-3β Phosphorylation in LPS-Stimulated MLE-12 Alveolar Epithelial Cells

**Figure 5:**
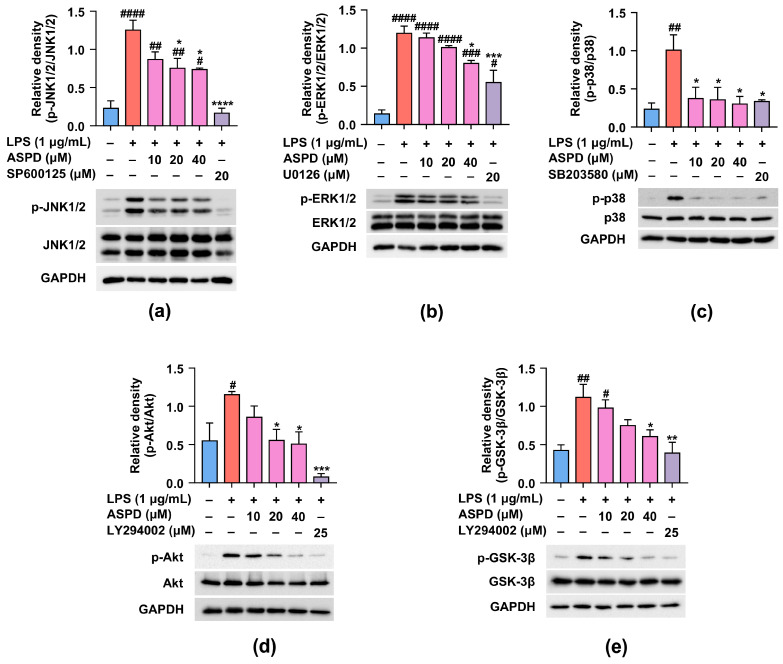
Effects of ASPD on MAPK and Akt/GSK-3β signalling pathways in LPS-stimulated MLE-12 cells. (**a**) JNK1/2; (**b**) ERK1/2; (**c**) p38 MAPK; (**d**) Akt; (**e**) GSK-3β protein expression levels were evaluated by Western blot analysis. The upper panels show the relative levels of phosphorylated proteins (p-JNK1/2, p-ERK1/2, p-p38 MAPK, p-Akt, and p-GSK-3β), which were quantified and normalised to their corresponding total protein levels. The lower panels show representative immunoblot images. Data are presented as mean ± SEM of three independent experiments. Statistical significance is indicated as follows: # *p* < 0.05, ## *p* < 0.01, ### *p* < 0.001, #### *p* < 0.0001 vs. the untreated control; * *p* < 0.05, ** *p* < 0.01, *** *p* < 0.001, **** *p* < 0.0001 vs. the LPS-treated group.

Based on the network pharmacology analysis and the established roles of MAPK and Akt/GSK-3β signalling in inflammatory responses and acute lung injury [13,15,17], selected hub targets and related signalling molecules were evaluated experimentally. Therefore, the effects of ASPD on the phosphorylation of ERK1/2, c-Jun N-terminal kinase (JNK1/2), p38 MAPK, Akt, and GSK-3β were examined in LPS-stimulated MLE-12 cells. ASPD treatment (20–40 μM) significantly reduced the phosphorylation of JNK1/2 (Figure 5a). Similarly, ASPD at 40 μM decreased extracellular signal-regulated kinase 1/2 (ERK1/2) phosphorylation (Figure 5b), whereas all tested concentrations reduced p38 MAPK phosphorylation (Figure 5c). Consistent with these findings, the specific inhibitors SP600125 (JNK inhibitor), U0126 (ERK1/2 inhibitor), and SB203580 (p38 MAPK inhibitor) also reduced phosphorylation of their respective targets (Figure 5).

In addition, ASPD (20–40 μM) reduced Akt phosphorylation, whereas 40 μM ASPD decreased GSK-3β phosphorylation (Figure 5d,e). The PI3K inhibitor LY294002 similarly reduced Akt and GSK-3β phosphorylation. Collectively, these results suggest that ASPD attenuates inflammatory responses in LPS-stimulated MLE-12 cells and is associated with alterations in MAPK and Akt/GSK-3β signalling. These findings are consistent with the observed reduction in pro-inflammatory cytokine production. Notably, several of these signalling proteins were identified as potential targets in the network pharmacology analysis, further supporting their potential involvement in the biological activity of ASPD.

### 2.4. ASPD Attenuates LPS-Induced Lung Injury and Pulmonary Oedema

**Figure 6:**
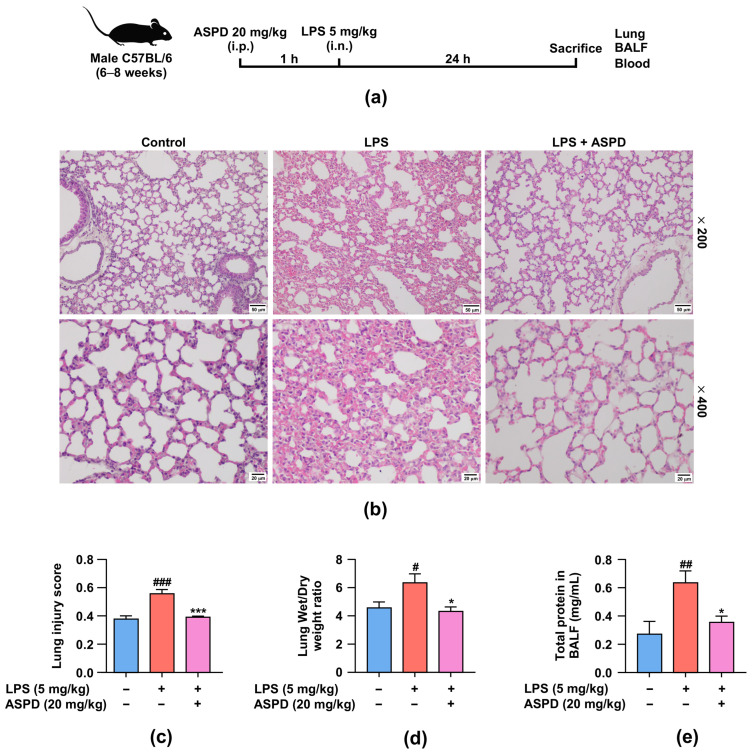
Effects of ASPD on histopathological changes and lung injury parameters in LPS-induced acute lung injury (ALI) mice. (**a**) Schematic diagram of the experimental design showing ASPD pretreatment (20 mg/kg, intraperitoneally) followed by LPS administration (5 mg/kg, intranasally) and sample collection after 24 h; (**b**) Representative histological images of lung tissues from each group stained with haematoxylin and eosin (H&E) (original magnification ×200 and ×400; scale bars = 50 µm and 20 µm, respectively); (**c**) Lung injury scores; (**d**) lung wet-to-dry weight ratio and (**e**) total protein concentration in bronchoalveolar lavage fluid (BALF). Data are presented as mean ± SEM (*n* = 5 per group). Statistical significance is indicated as follows: # *p* < 0.05, ## *p* < 0.01, ### *p* < 0.001 vs. control; * *p* < 0.05, *** *p* < 0.001 vs. the LPS-treated group.

To evaluate the protective effect of ASPD, an LPS-induced ALI mouse model was employed. Mice were administered ASPD (10, 20, or 40 mg/kg, intraperitoneally) 1 h prior to intranasal LPS challenge. Based on preliminary dose-ranging experiments, 20 mg/kg was selected for subsequent studies because it provided the most favourable improvement in ALI-related parameters without evidence of systemic toxicity, as assessed by the measured markers (Figure S1). Lung injury was evaluated after 24 h (Figure 6a).

LPS challenge induced severe lung injury characterised by marked histopathological alterations and pulmonary oedema. Histological analysis using H&E staining revealed prominent inflammatory cell infiltration, alveolar wall thickening, and disruption of alveolar structures in LPS-treated mice (Figure 6b). In contrast, ASPD pretreatment attenuated these pathological changes and preserved lung architecture. Consistently, ASPD significantly reduced the ALI score compared with the LPS group (Figure 6c). Moreover, ASPD significantly attenuated pulmonary oedema, as evidenced by a decreased lung wet-to-dry (W/D) weight ratio and reduced protein concentration in bronchoalveolar lavage fluid (BALF) (Figure 6d,e). These results indicate that ASPD mitigates LPS-induced lung injury and oedema in mice.

### 2.5. ASPD Attenuates Local Pulmonary and Systemic Inflammatory Responses in LPS-Induced ALI

**Figure 7:**
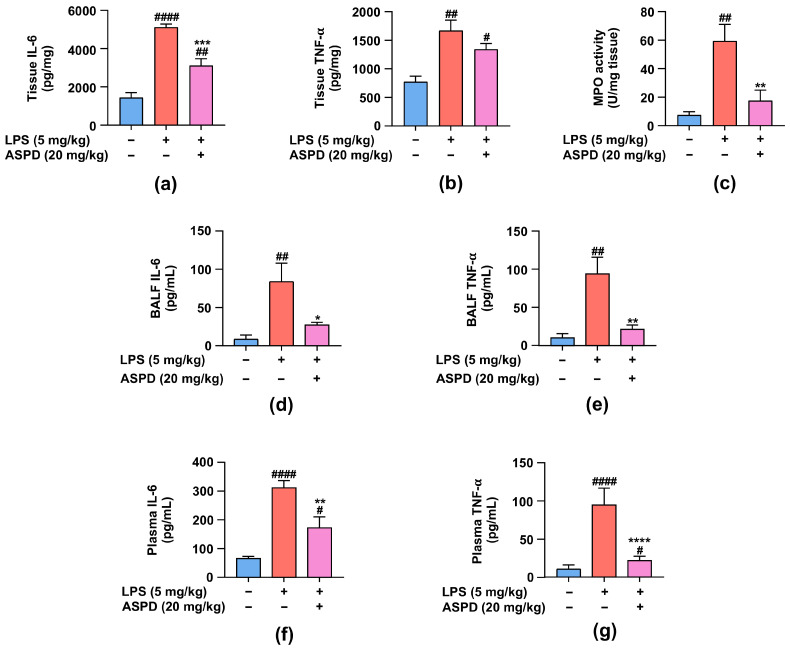
Effects of ASPD on inflammatory cytokines and myeloperoxidase (MPO) activity in LPS-induced ALI mice. Lung tissues were homogenised and the supernatants were used to determine (**a**) IL-6, (**b**) TNF-α, and (**c**) MPO activity. BALF was collected to measure (**d**) IL-6 and (**e**) TNF-α levels. Plasma samples were used to determine (**f**) IL-6 and (**g**) TNF-α levels, which are associated with systemic inflammatory responses. Data are presented as mean ± SEM (*n* = 5 per group). Statistical significance is indicated as follows: # *p* < 0.05, ## *p* < 0.01, #### *p* < 0.0001 vs. control; * *p* < 0.05, ** *p* < 0.01, *** *p* < 0.001, **** *p* < 0.0001 vs. the LPS-treated group.

To assess inflammatory responses in the ALI model, inflammatory cytokines were measured in lung tissue, BALF, and plasma. LPS challenge significantly increased IL-6 and tumour necrosis factor-alpha (TNF-α) levels across all compartments (Figure 7). In lung tissue, ASPD treatment significantly reduced IL-6 levels, whereas TNF-α levels showed a decreasing trend but did not reach statistical significance (Figure 7a,b). In addition, myeloperoxidase (MPO) activity, a marker of inflammatory cell infiltration, was markedly increased following LPS administration and was significantly reduced by ASPD treatment (Figure 7c). In BALF, ASPD significantly decreased both IL-6 and TNF-α levels compared with the LPS group (Figure 7d,e). Similarly, in plasma, ASPD significantly reduced IL-6 and TNF-α levels (Figure 7f,g). These results indicate that ASPD attenuates both local pulmonary and systemic inflammation in mice with LPS-induced ALI.

### 2.6. ASPD Reduces Src and ERK1/2 Immunoreactivity in Lung Tissues of LPS-Induced ALI Mice

**Figure 8:**
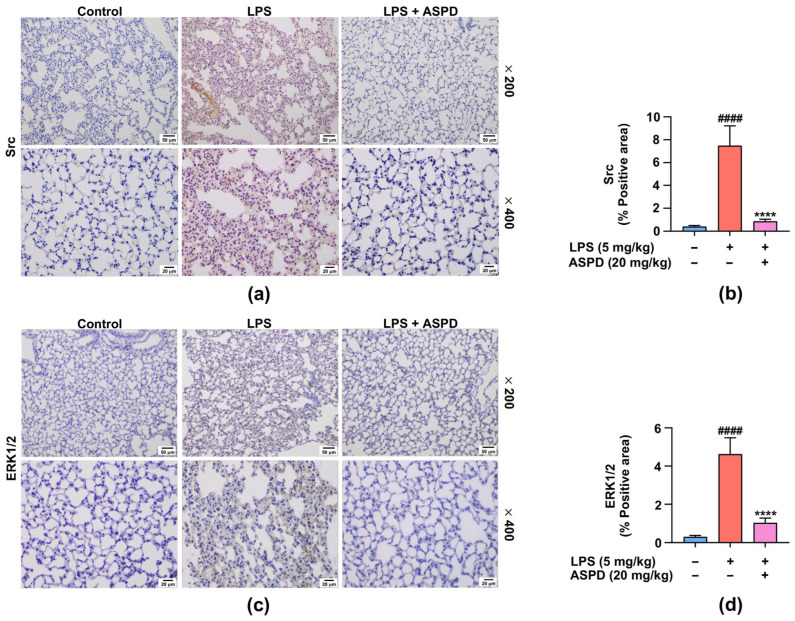
Effects of ASPD on Src and ERK1/2 immunoreactivity in LPS-induced ALI mice. Representative immunohistochemical staining of (**a**) Src and (**c**) ERK1/2 in lung tissues from each treatment group (original magnification ×200 and ×400; scale bars = 50 µm and 20 µm, respectively). Quantification of the percentage of positive staining area for (**b**) Src and (**d**) ERK1/2. For each mouse, three random fields per section were analysed at ×400 magnification, and a total of 15 images per group were quantified using ImageJ software. The intensity of brown staining reflects the immunoreactivity of the target proteins. Data are presented as mean ± SEM (*n* = 5 per group). Statistical significance is indicated as follows: #### *p* < 0.0001 vs. control; **** *p* < 0.0001 vs. the LPS-treated group.

Among the hub targets identified through network pharmacology analysis, Src and MAPK1 (ERK2) were selected for further evaluation because they were among the highest-ranked nodes in the PPI network and have been implicated in inflammatory signalling associated with acute lung injury. Given the established role of ERK signalling in LPS-induced acute lung inflammation [15], Src and ERK1/2 immunoreactivity were assessed in lung tissues from mice with LPS-induced acute lung injury. Compared with the control group, LPS challenge significantly increased Src and ERK1/2 immunoreactivity in lung tissues, as indicated by an increased percentage of positively stained area (Figure 8). ASPD treatment significantly reduced the percentage of Src-positive area (Figure 8a,b) and ERK1/2-positive area (Figure 8c,d) compared with the LPS group. These findings suggest that the protective effects of ASPD against LPS-induced ALI are associated with reduced Src and ERK1/2 immunoreactivity in lung tissues.

### 2.7. ASPD Shows No Apparent Adverse Effects on Selected Biochemical Parameters, Body Weight, and Organ Indices in LPS-Treated Mice

To evaluate the potential systemic toxicity of ASPD, plasma biochemical parameters related to liver and kidney function were measured. LPS challenge significantly increased aspartate aminotransferase (AST) and alanine aminotransferase (ALT) levels compared with the control group (Table 1). ASPD treatment significantly reduced AST and ALT levels compared with the LPS group. In contrast, lactate dehydrogenase (LDH) and creatinine levels were not significantly different among the groups.

Body weight and organ indices are presented in Table 2 and Table S6. No significant differences in body weight were observed among the groups. Similarly, organ indices were comparable across all groups. Collectively, these findings suggest that ASPD exhibited no apparent adverse effects on plasma biochemical parameters, body weight, or organ indices, indicating a favourable short-term safety profile under the experimental conditions examined.

### 2.8. Molecular Docking Analysis Reveals Potential Interactions Between ASPD and Selected Src- and MAPK-Related Signalling Proteins

To further investigate the potential interactions between ASPD and signalling proteins implicated in the pathways modulated in the present study, molecular docking analyses were performed with Src, JNK1/2, ERK1/2, and p38 MAPK as targets. These proteins were selected based on the experimental findings that ASPD reduced Src immunoreactivity in lung tissues and suppressed the phosphorylation of ERK1/2, JNK1/2, and p38 MAPK in LPS-stimulated MLE-12 cells.

**Figure 9:**
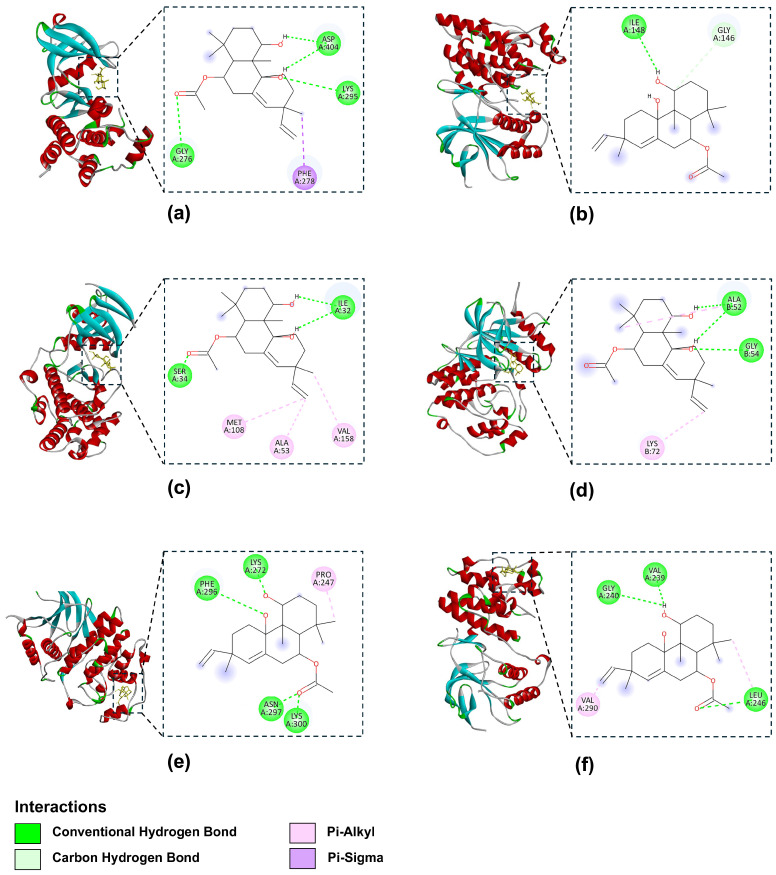
Three- and two-dimensional molecular docking models of ASPD with ALI-related targets. The binding interactions between ASPD and the targets are shown for (**a**) Src, (**b**) JNK1, (**c**) JNK2, (**d**) ERK1, (**e**) ERK2, and (**f**) p38 MAPK. Green dashed lines indicate hydrogen bonds, whereas pink or purple lines represent hydrophobic interactions.

The docking results demonstrated favourable binding affinities between ASPD and all selected targets, with binding energies ranging from −7.03 to −7.95 kcal/mol (Table 3). ASPD showed predicted binding energies of −7.63 kcal/mol for Src, −7.12 and −7.78 kcal/mol for JNK1 and JNK2, respectively, −7.03 kcal/mol for both ERK1 and ERK2, and −7.95 kcal/mol for p38 MAPK. Analysis of the docked complexes revealed that ASPD formed multiple hydrogen-bond and hydrophobic interactions with all selected targets. Hydrogen-bond interactions were observed with Src (GLY276, ASP404, and LYS295), JNK1 (ILE148 and GLY146), JNK2 (ILE32 and SER34), ERK1 (ALA52 and GLY54), ERK2 (PHE296, LYS272, ASN297, and LYS300), and p38 MAPK (VAL239, GLY240, and LEU246), while additional Pi–Alkyl or Pi–Sigma interactions were detected in each complex (Figure 9). These interactions may contribute to the favourable binding affinities observed for the selected signalling proteins. Overall, the docking analysis provided structural support for the potential interaction of ASPD with selected Src- and MAPK-related signalling proteins.

## 3. Discussion

Current therapeutic approaches for acute respiratory distress syndrome demonstrate variable clinical efficacy, highlighting the need for novel treatment strategies [5]. Plant-derived natural compounds have attracted increasing attention as potential multi-target anti-inflammatory agents for the treatment of acute lung injury [27]. In this study, ASPD, a major isopimarane-type diterpenoid, was evaluated for its protective effects against LPS-induced ALI through an integrative approach combining network pharmacology and molecular docking with in vitro and in vivo validation. Consistent with the predicted signalling targets and pathways identified by network pharmacology analysis, ASPD attenuated inflammatory responses and lung injury, as reflected by reduced cytokine production, ameliorated histopathological changes, and decreased pulmonary oedema. These effects were accompanied by reduced phosphorylation associated with MAPK and Akt/GSK-3β signalling in LPS-stimulated alveolar epithelial cells, together with reduced Src and ERK1/2 immunoreactivity in lung tissues. Molecular docking analysis further predicted favourable binding affinities between ASPD and selected Src- and MAPK-related signalling proteins, providing structural support for their potential interaction with ASPD.

Disruption of the alveolar–capillary barrier is a key feature of ALI and contributes to increased vascular permeability and pulmonary oedema [4]. ASPD significantly reduced the lung W/D ratio and BALF protein levels, suggesting attenuation of pulmonary oedema and vascular leakage, which may be associated with preservation of alveolar–capillary barrier function. These effects were accompanied by improved histopathological features, including reduced inflammatory cell infiltration and maintenance of alveolar structure. Excessive inflammation and immune cell infiltration are important contributors to ALI pathogenesis, and increased MPO activity is widely used as a marker of neutrophil infiltration in lung tissue [28]. The reductions in cytokine levels and MPO activity following ASPD treatment are consistent with attenuation of inflammatory responses associated with ALI, in which pro-inflammatory mediators and chemokines contribute to neutrophil recruitment and tissue injury [29]. Activated neutrophils release reactive oxygen species and neutrophil-derived proteases that contribute to lung injury [30].

The protective effects of ASPD observed in the present study are consistent with previous reports describing the anti-inflammatory and lung-protective activities of diterpenoids in experimental models of pulmonary injury. A recent study demonstrated that sphaeropsidin A, an isopimarane-type diterpenoid, attenuated LPS-induced acute pneumonia through modulation of the Keap1/Nrf2 signalling pathway [31]. Similar protective effects have also been reported for other diterpenoids. For instance, tanshinone IIA, an abietane-type diterpenoid, attenuated sepsis-induced lung injury by suppressing VEGFR2/PI3K/AKT-mediated mitochondrial dysfunction and apoptosis [32], whereas andrographolide protected against LPS-induced ALI through inhibition of NF-κB signalling, leading to reduced inflammatory cytokine production and pulmonary inflammation [33]. Similar to these diterpenoids, ASPD attenuated pulmonary inflammation and lung injury in the present study. Collectively, these findings indicate that diterpenoids exhibit lung-protective effects, although the underlying molecular targets and signalling pathways may differ.

ASPD is one of several structurally related isopimarane-type diterpenoids reported from *Kaempferia* species [25,34]. Congeners lacking an acetate ester have also been reported to exhibit anti-inflammatory activity, indicating that the acetate moiety is not an absolute requirement for biological activity. However, comparison of closely related analogues suggests that the introduction of a 6β-acetoxy substituent is associated with a notable increase in anti-inflammatory activity. Therefore, the potency of ASPD is likely influenced by the overall oxygenation pattern of the diterpenoid scaffold rather than by the acetate ester alone [34].

To further investigate signalling pathways associated with the protective effects of ASPD, its effects on key intracellular signalling proteins were evaluated in both in vitro and in vivo models. In LPS-stimulated MLE-12 cells, ASPD reduced the phosphorylation of MAPK- and Akt/GSK-3β-related signalling proteins, accompanied by reductions in pro-inflammatory cytokine levels. These signalling pathways are among the downstream inflammatory cascades associated with LPS–TLR4 signalling and regulate cytokine production in pulmonary epithelial cells [35]. MAPK signalling, particularly ERK1/2 and p38 MAPK, contributes to hyperoxia-induced inflammatory responses in MLE-12 cells and is associated with increased IL-6 production [36]. Similarly, p38 MAPK and NF-κB signalling pathways are involved in inflammatory responses in LPS-stimulated human alveolar epithelial A549 cells [37]. In addition, PI3K inhibition attenuates Akt and GSK-3β activation in LPS-stimulated MLE-12 cells, supporting a role for this pathway in inflammatory regulation [38]. The suppression of inflammatory cytokines by ASPD is associated with alterations in epithelial-associated inflammatory signalling. Since AT2 cells contribute to the regulation of alveolar inflammation during lung injury [39], the observed effects of ASPD in MLE-12 cells suggest a potential role in modulating epithelial inflammatory responses under inflammatory conditions. Consistent with the in vitro findings, ASPD treatment in LPS-induced ALI mice reduced Src and ERK1/2 immunoreactivity in lung tissues. Src kinase contributes to inflammatory responses through ERK1/2 signalling in experimental acute lung injury [40]. Modulation of MAPK signalling has been associated with attenuation of experimental lung injury in studies of plant-derived natural compounds [27]. The concurrent attenuation of MAPK and Akt/GSK-3β signalling observed in vitro, together with reduced Src and ERK1/2 immunoreactivity in vivo, suggests that the protective effects of ASPD may be associated with alterations in kinase signalling pathways. These molecular changes were accompanied by improvements in histopathological features, pulmonary oedema, and inflammatory cell infiltration, supporting a potential association between signalling alterations and the observed protective effects of ASPD.

Notably, network pharmacology analysis identified several inflammation-related hub targets, including Src, GSK-3β, ERK2, MEK1, and p38α MAPK, which are associated with MAPK and PI3K/Akt signalling. Enrichment analyses highlighted biological processes related to inflammatory and stress responses, both of which are implicated in ALI pathogenesis [41]. Molecular function enrichment indicated protein kinase activity, including serine/threonine and tyrosine kinases. These findings suggest the involvement of protein kinase signalling in inflammatory responses associated with ALI [40]. KEGG pathway analysis identified enrichment of HIF-1 signalling, chemokine signalling, and neutrophil extracellular trap formation. These pathways are relevant to ALI pathogenesis, including epithelial stress responses, chemokine-mediated inflammatory cell recruitment and NET-associated epithelial injury [42,43,44]. Consistent with these enrichment analyses, our experimental results demonstrated alterations in Src, MAPK, and Akt/GSK-3β signalling associated with ASPD treatment, supporting its potential role in attenuating ALI-associated inflammation. Although the network pharmacology analysis identified additional hub targets, including STAT3, TLR4, NFKB1, HIF1A, MMP9, and PTGS2, the present study focused on validating signalling molecules associated with the MAPK and Akt/GSK-3β pathways. Therefore, the potential contributions of these additional hub targets to the protective effects of ASPD remain to be elucidated and warrant further investigation. Although molecular docking supported potential interactions between ASPD and selected Src- and MAPK-related signalling proteins, docking provides only predictive evidence of ligand–target interactions and does not establish direct target engagement [45]. Therefore, additional mechanistic studies, including kinase activity assays, gene silencing approaches, pathway rescue experiments, target engagement analyses, proteomic approaches, and comparisons with selective pathway inhibitors, are warranted to further clarify the molecular targets and signalling mechanisms underlying the protective effects of ASPD. Nevertheless, integration of network pharmacology predictions, molecular docking analyses, and experimental validation suggests that ASPD may act through multiple interconnected signalling pathways to attenuate LPS-induced lung inflammation and injury, consistent with the concept of multi-target regulation proposed for bioactive natural compounds [46,47].

**Figure 10:**
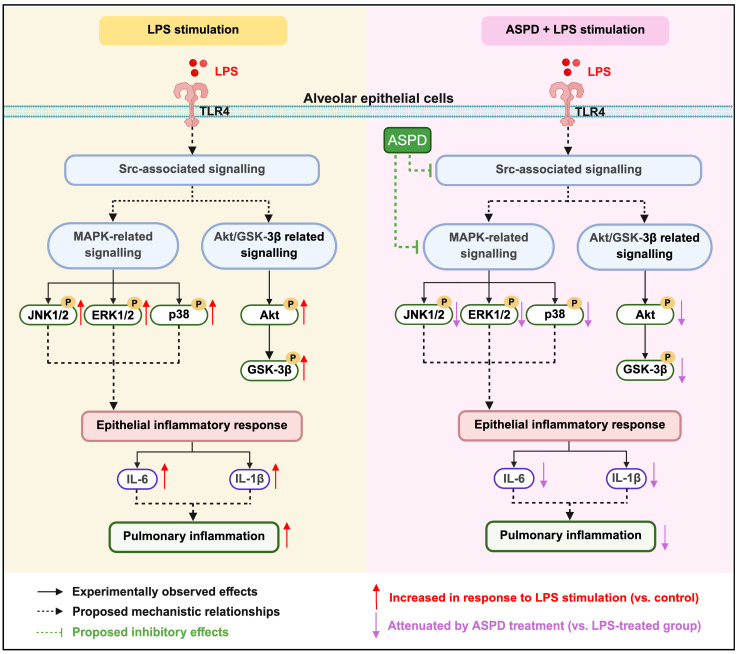
Proposed signalling network associated with the anti-inflammatory and protective effects of ASPD against LPS-induced ALI. The schematic illustrates the potential involvement of Src-, MAPK-, and Akt/GSK-3β-related signalling in inflammatory cytokine production, epithelial inflammatory responses, and pulmonary inflammation.

Based on these findings, a proposed signalling network associated with the anti-inflammatory and protective effects of ASPD against LPS-induced acute lung injury is presented in Figure 10. The model summarizes the potential involvement of Src, MAPK, and Akt/GSK-3β signalling in regulating inflammatory cytokine production, epithelial inflammatory responses, and pulmonary inflammation. By integrating experimentally observed molecular changes with signalling relationships reported in previous studies [15,17,40], the proposed network provides a conceptual framework for understanding the potential multi-target actions of ASPD. Nevertheless, the proposed interactions and pathway relationships remain hypothetical and require further mechanistic validation.

In addition to its protective effects against LPS-induced ALI, the short-term safety profile of ASPD was evaluated to assess potential systemic toxicity. LPS challenge increased plasma AST and ALT levels, suggesting mild hepatic involvement, consistent with previous evidence that pulmonary inflammation may contribute to lung–liver crosstalk [48]. ASPD reduced AST and ALT levels compared with the LPS group, suggesting attenuation of inflammation-associated hepatic responses and no apparent hepatotoxicity based on the biochemical parameters evaluated. LDH and creatinine levels remained unchanged, which did not suggest substantial systemic tissue injury or renal dysfunction under the experimental conditions examined. However, renal injury may occur without marked increases in serum creatinine during sepsis and related inflammatory conditions, partly due to reduced creatinine production [49]. Similarly, LDH is a nonspecific marker of cellular injury and may not fully reflect localized tissue damage [50]. Together with the absence of significant changes in body weight and organ indices, these findings suggest that ASPD was generally well tolerated during the study period. Nevertheless, the safety evaluation in the present study was limited to short-term assessments of biochemical parameters and organ indices in an acute LPS-induced ALI model. Comprehensive toxicological evaluation and long-term safety studies are warranted to further establish the safety profile of ASPD and support its future development.

This study provides a basis for further investigation of ASPD as a potential therapeutic candidate for ALI. A limitation of the present study is that the in vitro experiments were performed using a single cell line (MLE-12). Although this model is relevant to epithelial inflammation during acute lung injury [51], validation in additional pulmonary and immune cell models would help further establish the generalizability of the observed effects of ASPD. Furthermore, evaluation in macrophage models and epithelial–macrophage co-culture systems would provide additional insight into the effects of ASPD on multicellular inflammatory responses within the lung microenvironment. The in vivo findings were obtained in an acute LPS-induced model, and future evaluation in chronic, infectious, or clinically relevant models would strengthen the translational potential of ASPD. Although this acute LPS-induced model reproduces key inflammatory features of human ARDS, it does not fully recapitulate the clinical complexity of the disease, which is characterised by heterogeneous aetiologies, diverse comorbidities, and complex clinical management [4].

Another limitation of the present study is that the hydrolytic and metabolic stability of ASPD was not directly investigated. Since ASPD contains a 6β-acetoxy substituent [34], hydrolysis of the acetoxy ester moiety, particularly through carboxylesterase-mediated metabolism, cannot be excluded under physiological conditions [52]. Therefore, the relative contribution of the parent compound and any potential deacetylated metabolites to the observed anti-inflammatory effects remains unclear. Further pharmacokinetic and metabolite characterisation are warranted to evaluate the stability and biotransformation of ASPD. Taken together, while the present findings support the protective potential of ASPD, further mechanistic, preclinical, and clinical studies are required to establish its therapeutic efficacy and support its future development.

## 4. Materials and Methods

### 4.1. In Silico Prediction

#### 4.1.1. Network Pharmacology Analysis

Potential targets of ASPD were predicted using SwissTargetPrediction (http://swisstargetprediction.ch, accessed on 8 August 2023), SuperPred (https://prediction.charite.de, accessed on 8 August 2023), and SEA (https://sea.bkslab.org, accessed on 8 August 2023). Genes associated with acute lung injury (ALI) were retrieved from GeneCards (https://www.genecards.org/, accessed on 8 August 2023), OMIM (https://www.omim.org/, accessed on 8 August 2023), and GEO (https://www.ncbi.nlm.nih.gov/geo/, accessed on 8 August 2023) databases. Overlapping targets between ASPD-related targets and ALI-associated genes were identified using the jvenn platform (http://jvenn.toulouse.inra.fr/, accessed on 8 August 2023).

To explore the interactions among these targets, a protein–protein interaction (PPI) network was constructed using the STRING database (version 12.0; https://string-db.org, accessed on 8 August 2023) with a minimum confidence score of 0.4. The interaction data were exported in tab-separated values (TSV) format and visualised in Cytoscape (version 3.10.0; https://apps.cytoscape.org, accessed on 10 August 2023). Hub genes were identified using the cytoHubba plugin (version 0.1), and the top 20 genes were ranked according to their degree values.

Functional enrichment analysis was performed to investigate the biological significance of the identified targets. GO annotation, including biological process (BP), cellular component (CC) and molecular function (MF), as well as KEGG pathway enrichment analysis, was conducted using ShinyGO version 0.82 (http://bioinformatics.sdstate.edu/go/, accessed on 8 August 2023) and visualised using the bioinformatics platform (https://www.bioinformatics.com.cn/, accessed on 8 August 2023). The top 20 significantly enriched GO terms and KEGG pathways were selected. Only Homo sapiens genes were included, and a false discovery rate (FDR) < 0.05 was considered statistically significant.

#### 4.1.2. Molecular Docking Analysis

To evaluate the interactions between ASPD and selected ALI-related target proteins, molecular docking was performed using AutoDockTools 4.2. Target protein crystal structures were retrieved from the RCSB Protein Data Bank (PDB; https://www.rcsb.org/, accessed on 3 March 2026; PDB IDs in Table 3). The ASPD ligand structure was sourced from ChemSpider (ID: 76775071; https://www.chemspider.com, accessed on 1 September 2023) in .mol format, converted to PDB, and hydrogenated. Atomic charges were computed using the AM1-BCC algorithm under the AMBER force field, and the structure was energy-minimized in UCSF Chimera (version 1.10.19) via successive steepest descent and conjugate gradient steps.

Protein preparation involved removing water and non-protein molecules, adding polar hydrogens, and preserving key catalytic cofactors or metal ions. Prepared structures were converted to PDBQT format. Docking was conducted using the Lamarckian genetic algorithm over 50 runs with a population size of 200 [53,54]. The conformation exhibiting the lowest binding energy (ΔG_docking_; kcal/mol) was selected for analysis. Visualizations were generated using BIOVIA Discovery Studio Visualizer (Dassault Systèmes BIOVIA Corp., San Diego, CA, USA).

### 4.2. Isolation of 6β-Acetoxysandaracopimaradien-1α,9α-diol (ASPD)

The air-dried rhizomes (5.0 kg) of *K. marginata* were collected from the same location as previously reported [25]. The plant material was ground and sequentially macerated with hexane. After removal of the solvent under reduced pressure, 240.3 g of crude hexane extract was obtained. The extract was subjected to column chromatography, leading to the isolation of ASPD (Figure 1a). The compound was characterised using spectroscopic methods, including infrared (IR), ^1^H nuclear magnetic resonance (^1^H NMR), ^13^C NMR, as well as mass spectrometry, followed by comparison with previously published data [55]. The purity of the isolated compound exceeded 95%, as determined by thin-layer chromatography and NMR analysis.

### 4.3. In Vitro Experiments

#### 4.3.1. Cell Culture

The MLE-12 murine alveolar epithelial type II cell line (ATCC, Manassas, VA, USA) was cultured in Dulbecco’s Modified Eagle Medium/Ham’s F-12 (DMEM/F12) supplemented with 10% foetal bovine serum and 100 U/mL penicillin–streptomycin (all from HyClone, Cytiva, Marlborough, MA, USA). Cells were maintained in a humidified incubator at 37 °C with 5% CO_2_.

#### 4.3.2. Cell Viability Assay

Cell viability was determined using MTT assay (Sigma-Aldrich, St. Louis, MO, USA) in LPS-stimulated MLE-12 cells. Cells were seeded in 96-well plates at a density of 6.3 × 10^4^ cells/cm^2^ and incubated under standard culture conditions. After 24 h, cells were pretreated with ASPD (5–160 μM) for 1 h prior to stimulation with LPS (1 μg/mL; *Escherichia coli* O55:B5, Sigma-Aldrich) for 24 h. The culture medium was removed, and cells were incubated with 100 μL MTT solution (0.5 mg/mL) for 3 h at 37 °C. Formazan crystals were dissolved in 100 μL dimethyl sulphoxide, and absorbance was measured at 560 nm using a microplate reader (Thermo Fisher Scientific, Waltham, MA, USA). All experiments were performed in three independent experiments, each in triplicate.

#### 4.3.3. Enzyme-Linked Immunosorbent Assay (ELISA)

MLE-12 cells were seeded in 96-well plates at a density of 6.3 × 10^4^ cells/cm^2^. After reaching approximately 70–80% confluence (48 h), cells were pretreated with ASPD (10–40 μM) for 1 h prior to stimulation with LPS (1 μg/mL) for 24 h. Culture supernatants were collected, and the levels of IL-6 and IL-1β were determined using ELISA kits (PeproTech, Cranbury, NJ, USA) according to the manufacturer’s instructions. All experiments were performed independently three times.

#### 4.3.4. Western Blot Analysis

MLE-12 cells were seeded in 6-well plates at a density of 6.3 × 10^4^ cells/cm^2^ and incubated for 48 h. Cells were pretreated with ASPD (10–40 μM) or specific inhibitors, including SP600125 (20 μM), U0126 (20 μM), SB203580 (20 μM), and LY294002 (25 μM), for 1 h before stimulation with LPS (1 μg/mL) for 30 min. Cells were lysed in lysis buffer (Cell Signaling Technology, Danvers, MA, USA) supplemented with a protease inhibitor cocktail (MedChemExpress, Monmouth Junction, NJ, USA) and phenylmethylsulphonyl fluoride (PanReac AppliChem, Darmstadt, Germany). Total protein concentration was determined using a Pierce Detergent Compatible Bradford Assay Kit (Thermo Fisher Scientific, Rockford, IL, USA).

Equal amounts of protein (50 μg) were separated by sodium dodecyl sulfate–polyacrylamide gel electrophoresis (SDS-PAGE) and transferred onto polyvinylidene fluoride (PVDF) membranes (Bio-Rad Laboratories, Hercules, CA, USA). Membranes were blocked with 5% skimmed milk in Tris-buffered saline with 0.1% Tween-20 (TBST) and incubated overnight at 4 °C with primary antibodies against p-JNK1/2, JNK1/2, p-ERK1/2, ERK1/2, p-p38 MAPK, p38 MAPK, p-Akt, Akt, p-GSK-3β, GSK-3β, and GAPDH (1:500–1:1000; Cell Signaling Technology). Membranes were incubated with horseradish peroxidase (HRP)-conjugated secondary antibodies (1:2000; Cell Signaling Technology) for 1 h at room temperature. Protein bands were visualised using Forte ECL substrate (Merck Millipore, Burlington, MA, USA) and detected with a ChemiDoc Imaging System (Bio-Rad Laboratories). Band intensities were quantified using ImageJ software (version 1.54; National Institutes of Health, Bethesda, MD, USA). All experiments were performed independently three times.

### 4.4. In Vivo Experiments

#### 4.4.1. Experimental Design

Male wild-type C57BL/6 mice (6–8 weeks old, approximately 26–28 g) were obtained from Nomura Siam International Co., Ltd. (Bangkok, Thailand). Animals were acclimatised to the animal facility for one week prior to the initiation of experimental procedures. Animals had not undergone any previous experimental procedures prior to study initiation. Mice were housed in polypropylene cages under controlled environmental conditions (12 h light/dark cycle, 23 ± 2 °C and 55–60% relative humidity) with free access to standard laboratory chow and water. All experimental procedures were approved by the Walailak University Institutional Animal Care and Use Committee (Approval No. WU-ACUC-66011). A preliminary dose-range finding study was conducted to determine the appropriate ASPD dose for the main experiment. Based on observed changes in lung injury parameters and biochemical safety profiles (Figure S1), 20 mg/kg ASPD was selected for subsequent in vivo evaluation. No statistical analysis was performed in this preliminary study.

Animals were randomly assigned to experimental groups using a computer-generated random number sequence. The experimental unit was an individual mouse. A total of 30 mice were allocated to three groups (*n* = 10 per group): control (vehicle + saline), LPS (vehicle + LPS), and ASPD-treated (ASPD + LPS). Because BALF collection and tissue-based analyses could not be performed on the same lung samples, mice were divided into two independent experimental sets (*n* = 15 per set; *n* = 5 per group). In the first set, mice were used for BALF collection for cytokine and biochemical analyses. In the second set, mice were used for tissue-based analyses. The sample size for each experimental set was determined using the Resource Equation Approach described by Arifin and Zahiruddin [56]. Each set comprised 15 mice distributed across three groups (*n* = 5 per group), yielding an E value of 12 (E = N − G, where N is the total number of animals and G is the number of groups), which is within the recommended range for animal experiments. Predefined inclusion criteria included healthy animals without visible signs of illness or injury at the start of the experiment. Animals that died before the planned experimental endpoint were excluded from subsequent analyses. These inclusion and exclusion criteria were established prior to the initiation of the study. No animals, samples, or data points met the exclusion criteria and therefore all animals were included in the final analyses. Investigators were aware of group allocation during animal randomisation and treatment administration. Histopathological evaluation of H&E-stained lung sections was performed by three investigators blinded to treatment allocation. Other outcome assessments and data analyses were conducted without blinding.

Mice were intraperitoneally injected with either vehicle or ASPD (20 mg/kg). One hour later, acute lung injury was induced by intranasal administration of LPS (5 mg/kg; *Escherichia coli* O55:B5, Sigma-Aldrich) in a total volume of 50 µL. After 24 h, mice were anaesthetised by intraperitoneal injection of Zoletil 100 (100 mg/kg) and then euthanised.

The primary outcome measure was the histopathological lung injury score assessed in H&E-stained lung sections collected 24 h after LPS administration. For sample collection, BALF was collected for the determination of inflammatory cytokine levels and protein concentration in the first experimental set. In the second experimental set, the upper right lung lobes were collected for MPO activity assays and cytokine measurements, whereas the lower right lung lobes were fixed in 10% neutral buffered formalin (Bio-Optica Milano S.p.A., Milan, Italy) for H&E staining and IHC. The left lung was used to determine the lung W/D ratio. Blood samples were collected via cardiac puncture, and major organs (brain, heart, kidney, liver, spleen, and stomach) were harvested to calculate organ index values.

#### 4.4.2. Histopathological Staining and Lung Injury Score Assessment

To evaluate lung injury, lung tissues were fixed in 10% neutral buffered formalin (Bio-Optica Milano S.p.A.), dehydrated and embedded in paraffin. Paraffin-embedded tissues were sectioned at 4 μm using a Microm HM325 rotary microtome (Thermo Fisher Scientific, Walldorf, Germany). Sections were stained with H&E (Leica Biosystems, Wetzlar, Germany) and examined using an Olympus BX43 light microscope (Olympus Corporation, Tokyo, Japan).

Lung injury was evaluated in a blinded manner according to previously described criteria [6], including neutrophil infiltration in the alveolar and interstitial spaces, hyaline membrane formation, proteinaceous debris in the airspaces and alveolar septal thickening, using a 0–2 scoring system. For each mouse, 10 randomly selected fields were independently assessed by three blinded investigators, and the scores were averaged to obtain a single value per animal. The average score per mouse was used for statistical analysis, with each mouse considered an independent biological replicate.

#### 4.4.3. Lung Wet-to-Dry Weight Ratio

To assess pulmonary oedema, the lung W/D ratio was determined. The left lung was excised and immediately weighed to obtain the wet weight. The tissue was then dried in an oven at 60 °C for 72 h until a constant weight was achieved. The dried lung was reweighed, and the W/D ratio was calculated.

#### 4.4.4. Collection, Preparation and Biochemical Analysis of BALF, Plasma, and Lung Tissues

At 24 h after LPS administration, BALF was collected by three intratracheal instillations of 0.8 mL ice-cold phosphate-buffered saline containing 0.5 mM ethylenediaminetetraacetic acid. The recovered BALF was centrifuged at 300× *g* at 4 °C for 10 min, and the cell-free supernatant was collected for protein and cytokine analyses. Blood samples were collected in heparinised tubes and centrifuged at 1500× *g* for 10 min at room temperature to obtain plasma. Lung tissues were homogenised using an IKA homogeniser (IKA-Werke GmbH & Co. KG, Staufen, Germany) in T-PER Tissue Protein Extraction Reagent containing Halt Protease Inhibitor Cocktail (Thermo Fisher Scientific). The homogenates were centrifuged at 12,000× *g* for 10 min at 4 °C, and the supernatants were collected for cytokine analyses.

Total protein levels in BALF were determined using a bicinchoninic acid (BCA) protein assay (Thermo Fisher Scientific). Cytokine levels (IL-6 and TNF-α) in BALF, plasma and lung tissue homogenates were quantified using ELISA kits (PeproTech) according to the manufacturer’s instructions.

#### 4.4.5. Myeloperoxidase Activity

MPO activity was measured as an indicator of neutrophil infiltration using a commercial assay kit (Sigma-Aldrich). Lung tissues were weighed and homogenised in assay buffer provided with the kit, supplemented with Halt Protease Inhibitor Cocktail. The homogenates were centrifuged at 12,000× *g* for 10 min at 4 °C to remove debris, and the resulting supernatants were used for MPO activity determination according to the manufacturer’s instructions. MPO activity was expressed as U/mg tissue.

#### 4.4.6. Immunohistochemistry

Protein expression in lung tissues was evaluated by IHC using a commercial staining kit (Elabscience Biotechnology Inc., Houston, TX, USA). Paraffin-embedded tissue sections were dewaxed and subjected to antigen retrieval in buffer for 15 min, followed by washing with TBST. Endogenous peroxidase activity was blocked using peroxidase blocking buffer for 15 min. Sections were incubated overnight at 4 °C with primary antibodies against ERK (1:150) and Src (1:300) (Cell Signaling Technology) in a humidified chamber. After incubation, sections were incubated with HRP-conjugated secondary antibodies and visualised using 3,3′-diaminobenzidine (DAB), followed by counterstaining with haematoxylin. Three non-overlapping fields per slide from each mouse were randomly selected and examined under a light microscope (Olympus Corporation) at magnifications of ×200 and ×400. The area of positive immunostaining was quantified using ImageJ software (version 1.54; National Institutes of Health).

#### 4.4.7. Plasma Biochemical Markers and Organ Indices

Biochemical markers of liver and kidney injury, including ALT, AST, LDH, and creatinine, were determined in plasma using an automated clinical chemistry analyser (Alinity ci-series analyser, Abbott Laboratories, Abbott Park, IL, USA). Major organs (brain, heart, kidney, liver, spleen and stomach) were excised and immediately weighed after euthanasia, and organ index values were calculated as [(organ weight/body weight) × 100]. Plasma biochemical markers and organ index values were evaluated as indicators of organ injury, hypertrophy or atrophy associated with LPS-induced injury and ASPD treatment.

### 4.5. Statistical Analysis

Data are expressed as mean ± standard error of the mean (SEM). Data normality was assessed using the Shapiro–Wilk test. When data were normally distributed, differences among groups were analysed using one-way ANOVA followed by Dunnett’s post hoc test. Data that did not meet the assumption of normality were analysed using the Kruskal–Wallis test followed by Dunn’s multiple comparisons test. All statistical analyses were performed using GraphPad Prism 9 (version 9.5.1; GraphPad Software, San Diego, CA, USA). A *p* value < 0.05 was considered statistically significant.

## 5. Conclusions

ASPD shows potential for further development as a therapeutic candidate for ALI and may exert its protective effects through mechanisms associated with inflammatory signalling pathways. The combined in vitro and in vivo findings suggest that ASPD may attenuate pulmonary inflammation and tissue injury while being generally well tolerated under the present experimental conditions. Integration of network pharmacology analysis, molecular docking predictions, and experimental findings further supports a potential multi-target mode of action involving interconnected inflammatory signalling pathways. These findings provide a basis for further investigation of ASPD, including studies to further clarify its molecular targets, pharmacokinetic properties, long-term safety, and translational potential.

## Figures and Tables

**Table 1:** Effects of ASPD on biochemical toxicity parameters in LPS-induced mice.

Parameters	Control	LPS	LPS + ASPD
AST (U/L)	73.40 ± 9.61	103.80 ± 5.18 #	69.40 ± 6.31 *
ALT (U/L)	19.40 ± 1.54	24.40 ± 0.51 #	17.00 ± 0.84 ***
LDH (U/L)	181.00 ± 19.91	228.00 ± 15.48	179.60 ± 17.95
Creatinine (mg/dL)	0.07 ± 0.01	0.08 ± 0.01	0.07 ± 0.01

Data are expressed as mean ± SEM (*n* = 5 per group). # *p* < 0.05 vs. the control group; * *p* < 0.05 and *** *p* < 0.001 vs. the LPS-treated group.

**Table 2:** Effects of ASPD on organ indices in LPS-induced mice.

Organ Index (%)	Control	LPS	LPS + ASPD
Brain	1.58 ± 0.04	1.56 ± 0.02	1.62 ± 0.02
Heart	0.49 ± 0.01	0.49 ± 0.01	0.50 ± 0.01
Kidney	1.24 ± 0.02	1.18 ± 0.03	1.20 ± 0.02
Liver	5.16 ± 0.09	5.29 ± 0.07	5.07 ± 0.14
Spleen	0.24 ± 0.01	0.25 ± 0.01	0.24 ± 0.01
Stomach	0.89 ± 0.05	1.06 ± 0.07	1.04 ± 0.07

Data are expressed as mean ± SEM (*n* = 10 per group). Organ index (%) = (organ weight/final body weight) × 100. No statistically significant differences were observed among the groups.

**Table 3:** Molecular docking results of ASPD with Src and selected MAPK-family proteins.

Proteins	PDB ID	Binding Energy (kcal/mol)	Ki (µM)	Amino Acid Residues in H-Bond Interaction (≤4 Å)
Src	2BDJ	−7.63	2.55	GLY276, LYS295, ASP404
JNK1	3PZE	−7.12	6.07	ILE148
JNK2	8ELC	−7.78	1.99	ILE32, SER34
ERK1	2ZOQ	−7.03	7.01	ALA52, GLY54
ERK2	1TVO	−7.03	7.07	LYS272, PHE296, ASN297, LYS300
p38 MAPK	1KV2	−7.95	1.49	VAL239, GLY240, LEU246

Abbreviations: Å, angstrom; PDB, Protein Data Bank; Ki, predicted inhibition constant. Lower binding energy and Ki values indicate stronger predicted protein–ligand interactions.

## Data Availability

The data presented in this study are available within the article and Supplementary Materials.

## Supplementary Materials

The following supporting information can be downloaded at: https://www.mdpi.com/article/10.3390/ijms27135969/s1.
