# Supplementary material for: 6β-Acetoxysandaracopimaradien-1α,9α-diol Attenuates LPS-Induced Acute Lung Injury: Association with Alterations in Src, MAPK, and Akt/GSK-3β Signalling
Source: Int J Mol Sci. 2026 Jul 3;27(13):5969. doi: 10.3390/ijms27135969 (PMC13362311; doi:10.3390/ijms27135969)
Supplement: Supplementary file 1 [file ijms-27-05969-s001.zip › ijms-4380402-supplementary.pdf]

Supplementary Data

6β-Acetoxysandaracopimaradien-1α,9α-diol Attenuates LPS-Induced Acute Lung Injury: Association with Alterations in Src, MAPK, and Akt/GSK-3β Signalling

Nassareen Supaweera [1], Wanatsanan Chulrik [2,3], Chutima Jansakun [2,3], Aman Tedasen [2], Chuchard Punsawad [4], Porawan Pratumwan [1], Rungruedee Kimseng [5], Ratchanaporn Chokchaisiri [6], Apichart Suksamrarn [7] and Warangkana Chunglok [2,3,*]

1 Health Sciences (International Program), College of Graduate Studies, Walailak University, Nakhon Si Thammarat 80160, Thailand
2 School of Allied Health Sciences, Walailak University, Nakhon Si Thammarat 80160, Thailand
3 Food Technology and Innovation Research Center of Excellence, Walailak University, Nakhon Si Thammarat 80160, Thailand
4 School of Medicine, Walailak University, Nakhon Si Thammarat 80160, Thailand
5 Sanders Brown Center on Aging, University of Kentucky College of Medicine, Lexington, KY 40536, USA
6 Department of Chemistry, School of Sciences, University of Phayao, Phayao 56000, Thailand
7 Department of Chemistry and Center of Excellence for Innovation in Chemistry, Faculty of Science, Ramkhamhaeng University, Bangkok 10240, Thailand
* Correspondence: cwarang@wu.ac.th

Table S1. Top 20 hub targets in the PPI network ranked by degree centrality. Targets with identical degree values share the same rank.

Rank	Name	Score
1	SRC	96
1	EGFR	96
3	STAT3	92
4	TLR4	84
4	NFKB1	84
6	HIF1A	80
7	MMP9	76
7	PTGS2	76
9	STAT1	72
10	GSK3B	66
11	MTOR	64
12	JAK2	62
12	MAPK1	62
14	MAP2K1	52
15	MAPK14	50
16	NFE2L2	48
16	PARP1	48
16	JAK1	48
19	PIK3CG	46
19	PTPN11	46

A**bbreviations:** EGFR, epidermal growth factor receptor; GSK3B, glycogen synthase kinase-3 beta; HIF1A, hypoxia-inducible factor 1-alpha; JAK1, Janus kinase 1; JAK2, Janus kinase 2; MAP2K1, mitogen-activated protein kinase kinase 1; MAPK1, mitogen-activated protein kinase 1; MAPK14, mitogen-activated protein kinase 14; MMP9, matrix metalloproteinase-9; MTOR, mammalian target of rapamycin; NFE2L2, nuclear factor erythroid 2-related factor 2; NFKB1, nuclear factor kappa B subunit 1; PARP1, poly [ADP-ribose] polymerase 1; PIK3CG, phosphatidylinositol 4,5-bisphosphate 3-kinase catalytic subunit gamma; PTGS2, prostaglandin-endoperoxide synthase 2; PTPN11, tyrosine-protein phosphatase non-receptor type 11; SRC, proto-oncogene tyrosine-protein kinase Src; STAT1, signal transducer and activator of transcription 1; STAT3, signal transducer and activator of transcription 3; TLR4, toll-like receptor 4.

Table S2. Gene Ontology (GO) of biological process enrichment analysis

No.	Term	Enrichment score	Gene count	Fold enrichment
1	Cellular response to chemical stimulus	32.04	54	6.89
2	Response to stress	31.19	65	4.38
3	Response to oxygen-containing compound	29.70	47	8.12
4	Inflammatory response	28.43	38	11.90
5	Response to chemical	28.37	62	4.30
6	Cellular response to oxygen-containing compound	28.20	41	9.83
7	Response to external stimulus	27.28	52	5.78
8	Defense response	25.55	47	6.42
9	Regulation of multicellular organismal process	25.49	54	4.93
10	Response to nitrogen compound	24.86	37	9.80
11	Locomotion	23.64	38	8.57
12	Intracellular signaling cassette	23.56	44	6.46
13	Regulation of response to external stimulus	23.48	37	8.89
14	Regulation of response to stress	23.41	40	7.61
15	Regulation of developmental process	22.49	47	5.40
16	Intracellular signal transduction	21.98	50	4.72
17	Regulation of cell differentiation	20.29	38	6.83
18	Regulation of biological quality	20.25	48	4.61
19	Cell migration	19.66	37	6.84
20	Cell motility	19.58	39	6.21

Table S3. Gene Ontology (GO) of cellular component enrichment analysis

No.	Term	Enrichment score	Gene count	Fold enrichment
1	Membrane raft	5.76	11	10.23
2	Membrane microdomain	5.76	11	10.17
3	Caveola	5.63	7	23.28
4	Cytoplasmic vesicle	5.33	28	2.91
5	Intracellular vesicle	5.33	28	2.91
6	Dendrite	4.98	13	5.94
7	Plasma membrane raft	4.98	7	16.61
8	Dendritic tree	4.98	13	5.92
9	Side of membrane	4.34	14	4.72
10	Cell junction	4.29	24	2.82
11	Phosphatidylinositol 3-kinase complex class ia	4.17	3	102.01
12	Neuron projection	4.17	17	3.66
13	Phosphatidylinositol 3-kinase complex class i	4.17	3	102.01
14	Plasma membrane region	4.17	17	3.67
15	Focal adhesion	4.04	10	6.25
16	Glutamatergic synapse	4.04	11	5.54
17	Cell-substrate junction	3.99	10	6.12
18	Somatodendritic compartment	3.91	13	4.41
19	Plasma membrane bounded cell projection	3.89	22	2.75
20	Cell surface	3.78	14	3.95

Table S4. Gene Ontology (GO) of molecular function enrichment analysis

No.	Term	Enrichment score	Gene count	Fold enrichment
1	Protein kinase activity	12.39	21	10.10
2	Phosphotransferase activity alcohol group as acceptor	12.36	22	8.96
3	Kinase activity	11.55	22	8.02
4	Catalytic activity acting on a protein	11.43	35	4.08
5	Transferase activity transferring phosphorus-containing groups	11.01	23	6.87
6	Adenyl nucleotide binding	10.05	28	4.72
7	Protein serine/threonine kinase activity	8.72	15	9.85
8	ATP binding	8.49	25	4.57
9	Adenyl ribonucleotide binding	8.30	25	4.45
10	Purine nucleotide binding	8.28	28	3.89
11	Protein tyrosine kinase activity	8.20	10	18.89
12	Transcription factor binding	8.03	16	7.56
13	Enzyme binding	8.03	28	3.73
14	Ribonucleotide binding	8.03	27	3.90
15	Protein serine kinase activity	8.03	13	10.69
16	Heterocyclic compound binding	7.76	29	3.50
17	Nucleotide binding	7.73	28	3.60
18	Nucleoside phosphate binding	7.68	28	3.57
19	Anion binding	7.32	29	3.32
20	Carbohydrate derivative binding	7.23	28	3.40

Table S5. Kyoto Encyclopedia of Genes and Genomes (KEGG) enrichment analysis

No.	Pathway	Enrichment score	Gene count	Fold enrichment
1	Pathways in cancer	23.70	28	16.20
2	Kaposi sarcoma-associated herpesvirus infection	22.09	20	31.55
3	PD-L1 expression and PD-1 checkpoint pathway in cancer	21.38	16	54.40
4	Human cytomegalovirus infection	19.52	19	25.96
5	Hepatitis B	18.91	17	31.92
6	HIF-1 signaling pathway	18.44	15	42.11
7	EGFR tyrosine kinase inhibitor resistance	16.93	13	50.36
8	Chemokine signaling pathway	16.38	16	25.77
9	Prolactin signaling pathway	15.87	12	51.72
10	Relaxin signaling pathway	15.87	14	33.21
11	Prostate cancer	15.87	13	41.01
12	Lipid and atherosclerosis	15.69	16	22.77
13	Neutrophil extracellular trap formation	15.05	15	24.16
14	Proteoglycans in cancer	14.65	15	22.61
15	AGE-RAGE signaling pathway in diabetic complications	14.18	12	37.09
16	Pancreatic cancer	13.77	11	43.72
17	Toxoplasmosis	13.61	12	33.08
18	Thyroid hormone signaling pathway	13.24	12	30.60
19	Growth hormone synthesis secretion and action	13.24	12	30.60
20	Chemical carcinogenesis-receptor activation	12.99	14	19.93

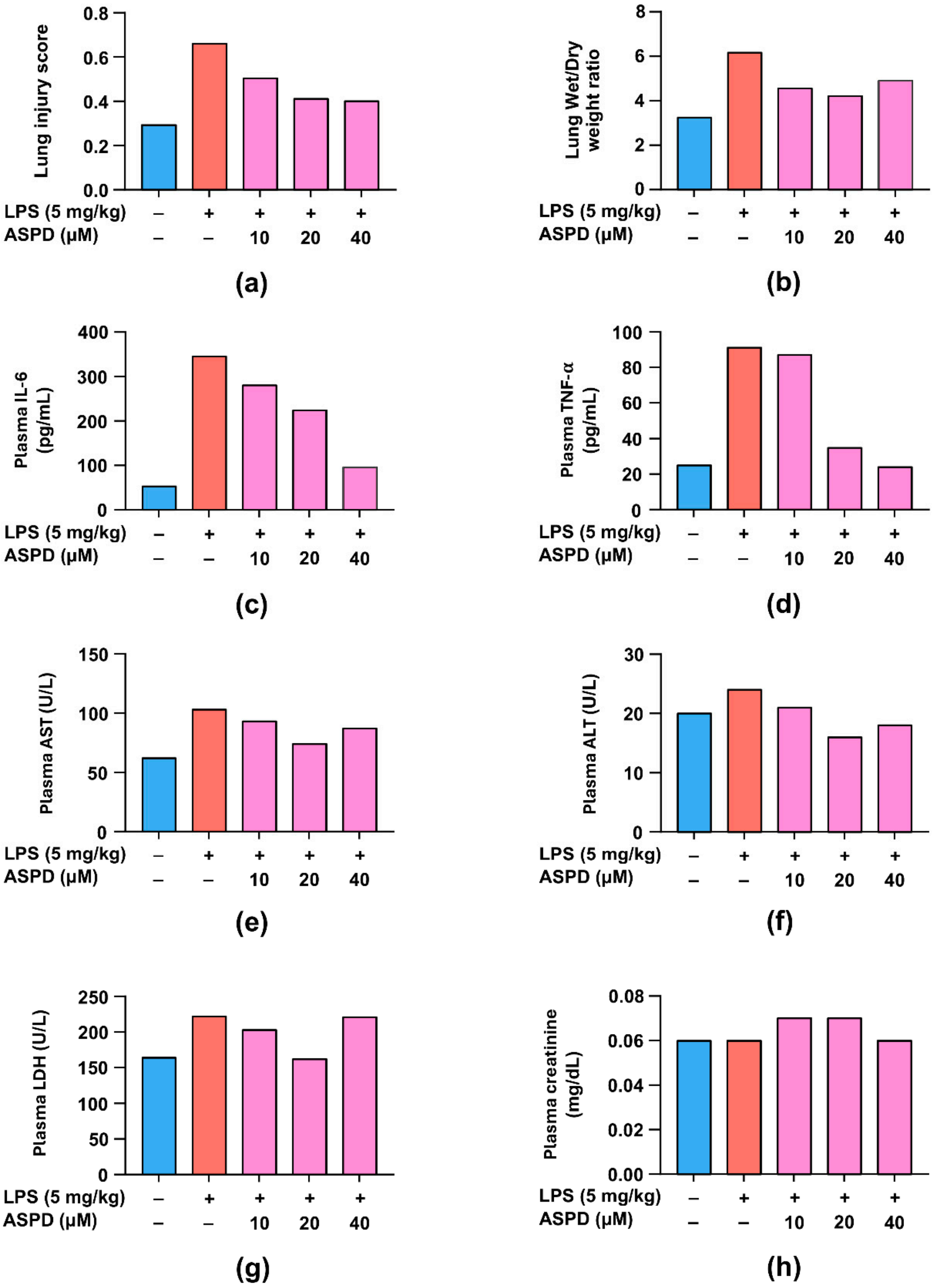

Figure S1. Preliminary dose-range-finding study of ASPD in LPS-induced acute lung injury (ALI). (**a**) Lung injury scores; (**b**) lung wet-to-dry weight ratio; (**c**) plasma IL-6; (**d**) plasma TNF-*α*; (**e**) plasma AST; (**f**) plasma ALT; (**g**) plasma LDH; and (**h**) plasma creatinine. Data are presented as individual values (*n* = 1 per group).

Table S6. Effects of ASPD on body weight and organ weight in LPS-induced mice.

**Parameters**	**Control**	**LPS**	**LPS + ASPD**
Body Weight (g)			
Initial weight	27.18 ± 0.91	28.17 ± 0.39	26.52 ± 0.29
Final weight	26.79 ± 0.92	27.53 ± 0.36	26.44 ± 0.36
Organ weight (g)			
Brain	0.42 ± 0.01	0.43 ± 0.00	0.43 ± 0.00
Heart	0.13 ±0.01	0.13 ± 0.00	0.13 ± 0.00
Kidney	0.33 ± 0.01	0.32 ± 0.01	0.32 ± 0.01
Liver	1.38 ± 0.06	1.46 ± 0.03	1.34 ± 0.04
Spleen	0.06 ± 0.00	0.07 ± 0.00	0.06 ± 0.00
Stomach	0.24 ± 0.02	0.29 ± 0.02	0.27 ± 0.02

Data are expressed as mean ± SEM (n = 10 per group).

Statistical analysis was performed using one-way ANOVA followed by Dunnett's post hoc test.

No statistically significant differences were observed among the groups.
